# Morphological and SERS Properties of Silver Nanorod Array Films Fabricated by Oblique Thermal Evaporation at Various Substrate Temperatures

**DOI:** 10.1186/s11671-015-0962-8

**Published:** 2015-06-10

**Authors:** Myoung-Kyu Oh, Yong-Seok Shin, Chang-Lyoul Lee, Ranjit De, Hoonsoo Kang, Nan Ei Yu, Bok Hyeon Kim, Joon Heon Kim, Jin-Kyu Yang

**Affiliations:** Advanced Photonics Research Institute (APRI), Gwangju Institute of Science and Technology (GIST), Gwangju, 500-712 South Korea; Department of Optical Engineering, Kongju National University, Cheonan, 330-717 South Korea

**Keywords:** Silver nanorod array, Oblique angle deposition, SERS, Thermal evaporation

## Abstract

Aligned silver nanorod (AgNR) array films were fabricated by oblique thermal evaporation. The substrate temperature during evaporation was varied from 10 to 100 °C using a home-built water cooling system. Deposition angle and substrate temperature were found to be the most important parameters for the morphology of fabricated films. Especially, it was found that there exists a critical temperature at ~90 °C for the formation of the AgNR array. The highest enhancement factor of the surface-enhanced Raman scattering (SERS), observed in the Ag films coated with benzenethiol monolayer, was ~6 × 10^7^. Hot spots, excited in narrow gaps between nanorods, were attributed to the huge enhancement factor by our finite-difference time-domain (FDTD) simulation reflecting the real morphology.

## Background

Enhancement of electromagnetic fields on metal nanostructures by surface plasmon excitation has received lots of attention for its great potential applications in highly sensitive chemical/bio-sensors [1] as well as effective optical devices [2, 3]. Surface-enhanced Raman scattering (SERS) is from the field enhancement mechanism and regarded as a powerful sensing tool since it can enable highly sensitive detection as well as multiple constituent analyses [1]. According to previous reports, the enhancement factor (E.F.) of the Raman signal in metal nanostructures is generally 10^4–7^ level, even though a value as high as 10^10–11^ was observed in the case of a special structure [4]. But SERS in a large area rather than in a specially prepared spot is needed for practical applications. Recently, various kinds of nanostructures with a large area, having E.F. higher than 10^8^, were introduced [5–7]. In such SERS-active media, trace even up to single molecular level detection is known to be possible without the help of another enhancement mechanism, such as resonant Raman scattering [8] and chemical enhancement [9].

For quantitative and repeated analysis, urgently needed in sensor technology, the substrate scheme is preferred over the solution one as the SERS sensor platform. So, there have been a number of methods introduced to fabricate novel SERS substrates until now, where the sensitivity or E.F. of the SERS media has always been the main issue. Deposition of chemically synthesized metal nanoparticles, such as spheres, rods, polyhedrons, stars, bonded or aggregated particles, shells, and bimetallic particles, on a flat surface has been the mainstream in the research field. Meanwhile, another important approach has been growing or carving metal nanostructures on substrates by physical or chemical deposition, lithography, anodic aluminum oxide (AAO) template method, etching, focused ion beam (FIB) method, etc. Out of all the products obtained through the aforementioned techniques, silver nanorod (AgNR) array film fabricated by oblique angle deposition (O.A.D.) method [5, 10] is thought to be the most promising SERS-active medium for its outstanding properties, such as large area, high uniformity, high productivity, and no chemical contaminants on the surface as well as the highest E.F. (10^8–9^) in a large area.

Metal nanorod array fabrication by O.A.D. method has been known for several decades [11, 12]. But the main usage as a SERS-active medium was from Zhao et al.’s work in 2005 [5], where the authors fabricated aligned AgNR array films by e-beam evaporation. When the deposition angle was larger than 80°, nanorods with diameter ~90 nm and density ~20 μm^−2^ were grown from the nuclei existing on the substrate in the early stage, to which the shadow effect is mainly attributed. The E.F. of the AgNR array with optimized morphology was reported as higher than 10^8^, which is the highest of the values observed in all kinds of SERS substrates of a large area until now. After the work, they have been making great efforts to explain the mechanism for the huge E.F. observed in the AgNR arrays [13–18] and improve the E.F. of those films [18, 19]. Applications to developing SERS bio-sensors have been the main interest of the authors [20]. Their works have been followed by other experiments [21, 22]. But the main contribution to the AgNR SERS film has been from the aforementioned team.

Even though much research has been conducted on the fabrication of AgNR arrays by e-beam evaporation and its applications in SERS, relatively little attention has been paid to fabricating AgNR arrays by mostly popular thermal evaporation [23, 24]. Singh et al. [24] reported the fabrication of AgNR array films by thermal evaporation using O.A.D. method. When the vapor incidence angle and the substrate temperature were 85° and 50 °C, respectively, well-separated AgNR arrays were fabricated in the experiment. However, any application as a SERS substrate was not conducted in the report. In addition, a systematic study about the effects of the substrate temperature on the morphology of the film has not been done until now. But substrate temperature is one of the most important parameters to influence the morphological properties of fabricated samples. Special attention should be paid to the substrate temperature during the fabrication of Ag films since the diffusion effect of Ag adatoms can play an important role even in a relatively low temperature range due to the low atomic energy barrier for the movement of adatoms. And heating by blackbody radiation from a high-temperature atomic source (tungsten boat) can increase the substrate temperature to a value as high as 100 °C like in this work where a thermal evaporator, having a bell-jar-type vacuum chamber, was employed.

In this work, we fabricated AgNR arrays by thermal evaporation at oblique angle, where the substrate temperature during evaporation was varied from 10 to 100 °C using a home-built water cooling system. The effect of the substrate temperature on the morphology of the samples was investigated by scanning electron microscopy (SEM). To reveal the mechanism for the AgNR formation, X-ray diffraction (XRD) analysis on the samples and a thermodynamic study on the diffusion effect of Ag adatoms were performed. The samples’ surfaces were functionalized by benzenethiol molecules for SERS. The morphology dependence of the E.F. was investigated, where the highest E.F. was as large as 10^8^ with the pump laser wavelength of 532 nm. To understand the underlying physics for the huge E.F. of the AgNR array, a theoretical study was performed based on numerical calculation by finite-difference time-domain (FDTD) method.

## Methods

**Fig. 1:**
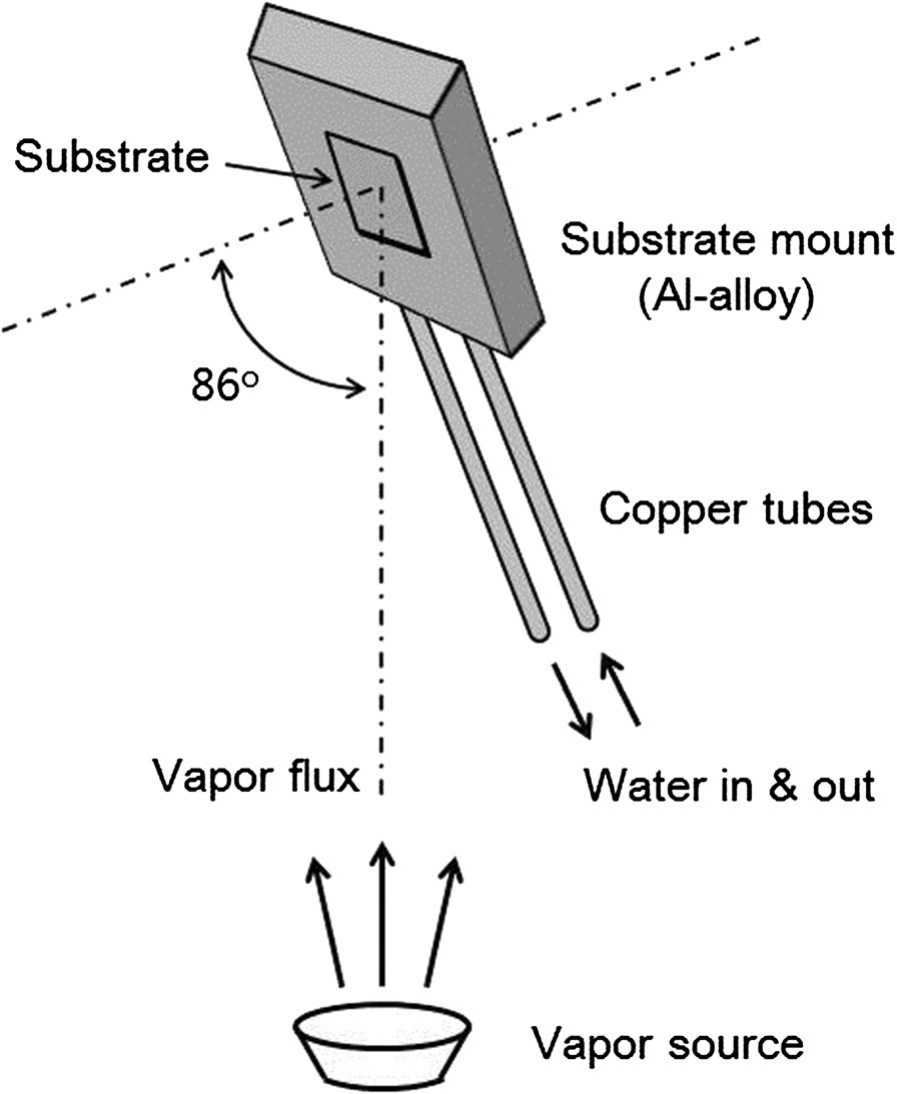
Schematic diagram of the substrate mount and cooling system

AgNR arrays were fabricated using O.A.D. method, where a bell-jar-type thermal evaporator (GV Tech., Inc.) was employed. For the evaporation, Ag powder (99.9 %) was used as the atomic source. The base pressure of the vacuum chamber was maintained below 5 × 10^−6^ Torr during the evaporation process. Figure 1 schematically shows the apparatus built in the evaporation chamber for the O.A.D. process. The substrate mount was set tilted for the deposition angle to be 86° ± 1°. The water flowing through the copper tubes controlled the substrate temperature. The temperature and the flow rate of the cooling water were controlled by a home-built controller. The copper tubes were bonded to the substrate mount made of aluminum alloy by welding. A temperature sensor (AD590, Thorlabs, Inc.) was installed in the substrate mount to monitor the substrate temperature during the evaporation process. Si wafers, used as deposition substrates, were placed on the center of the substrate mount. When the water cooling system did not work, the substrate temperature during the evaporation process was observed to increase up to 100 °C due to the blackbody radiation from the vapor source. But the substrate temperature could be controlled from 10 to 70 °C by the cooling system. The thickness of the deposited Ag film was monitored by a quartz crystal microbalance (QCM) directed normal to the vapor flux.

The morphologies of the deposited AgNR films were investigated by scanning electron microscopy (SEM). And the crystallographic structure of the AgNR arrays was characterized by X-ray diffraction analysis (XRD) using a computer-controlled Rigaku diffractometer with a Cu radiation (*λ* = 0.15406 nm) running at 40 kV and 40 mA.

The SERS properties of the AgNR array samples were measured by a home-built Raman spectrometer which employs a C-T spectrometer (Spectro, Inc.) with a resolution of 0.3 nm at 532 nm and a cooled Si-CCD detector (Andor, Inc., model name: iVac). As laser sources, continuous-wave (CW) diode lasers of 532, 633, and 785 nm were employed, so the wavelength of the pump laser could be selected from the wavelengths. The linewidths of the laser sources are smaller than 0.3 nm in full width at half maximum (FWHM). Kinds of optical filters were installed in the Raman spectrometer for all the wavelengths to be available in the SERS experiment. The pump laser incidence angle was normal to the substrate, and the collection angle of the Raman signal was also normal to the substrate.

The surfaces of the AgNR array films were functionalized by benzenethiol molecules for SERS. First, the Ag film samples were incubated in ethanoic solutions of benzenethiol (10 mM) for 4 h at room temperature in a glove box containing N_2_ gas. And then, the substrates were carefully rinsed with a copious amount of ethanol to make them free from any physically adsorbed thiol molecule, thereby resulting in the formation of self-assembled monolayers (SAMs). Finally, drying of the samples under the stream of N_2_ gas followed. The substrates functionalized in this way were then taken for SERS measurements.

## Results and Discussion

### Mechanism of AgNR Array Formation and the Role of Substrate Temperature

**Fig. 2:**
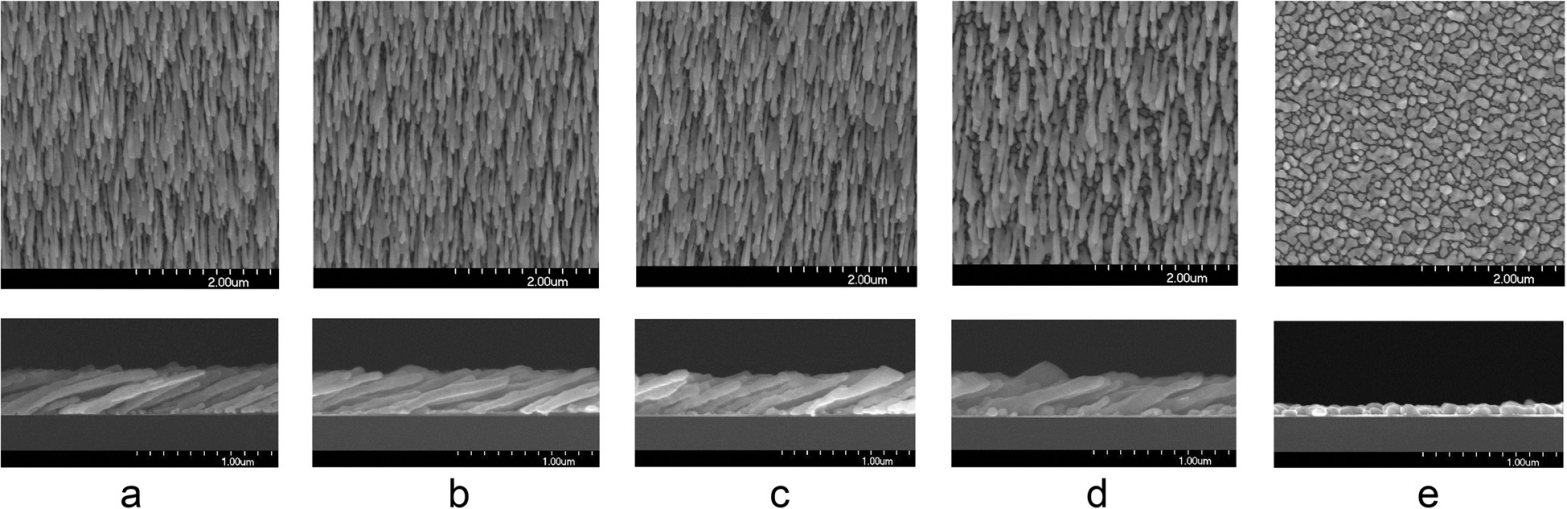
SEM images of Ag films fabricated at different substrate temperatures of (**a**) 10, (**b**) 30, (**c**) 50, (**d**) 70, and (**e**) 100 °C. *Scale bars* in the *upper images* correspond to 2 μm and those in the *lower images* 1 μm

Figure 2 shows the SEM images of the Ag nanostructures fabricated at various substrate temperatures (10 to 100 °C). The angle and the rate of deposition were 86° ± 1° and 0.5 nm/s for all the samples, respectively. But the deposition amount, monitored by the QCM, was 3 μm in (a)–(d) and 1 μm in (e). Figure 2a–d shows highly porous nanorod array structures, whose morphologies show strong dependence on the substrate temperature. The average diameters of the nanorods are 58 ± 7, 63 ± 7, 66 ± 7, and 79 ± 10 nm; the average lengths 860 ± 180, 840 ± 90, 770 ± 100, and 700 ± 70 nm; and the average densities 23 ± 2, 21 ± 2, 21 ± 2, and 13 ± 1 rods/μm^2^ at 10, 30, 50, and 70 °C, respectively. But the tilting angles are universally 70° ± 2° in (a)–(d). So to speak, the effect of substrate temperature on the morphology of the fabricated AgNR is that the rise of substrate temperature leads to an increase in diameter, but a decrease in length and density, while no apparent change in tilting angle within the above temperature range. Bundling between neighboring AgNRs is found in many sites in Fig. 2a, but it decreases fast as the substrate temperature increases. There are few bundling nanorods in Fig. 2d.

Except for the temperature dependence, the morphologies of the AgNR arrays fabricated by thermal evaporation are very similar to those of the samples by e-beam evaporation assuming the same deposition parameters [3]. However, when the substrate temperature was increased to 100 °C, the aforementioned porous nanorod array structure disappeared and a flat Ag film, consisting of a micrometer-size domain, appeared as shown in Fig. 2e. The coalescence and/or aggregation among neighboring nuclei, known as the bundling effect, seems to be much more prominent at this substrate temperature, resulting in the inhibition of columnar growth and the appearance of this bulk structure.

The above observations could be explained by two mechanisms, namely, shadow effect and diffusion effect. As well known, the shadow effect always plays important roles for the growth of nanorods in O.A.D. But the diffusion effect or adatoms’ mobility should have played a crucial role for the temperature dependence of the morphological properties observed in the AgNR arrays.

First, the influence of the diffusion effect appears in the tilting angle. The observed tilting angles of the AgNRs are much larger than those of the other metal nanorods observed in previous experiments assuming the same deposition angle and substrate temperature [11, 12]. This phenomenon was also observed by Zhao et al*.* [10]. The tilting angle, *β*, observed in their AgNR samples did not show any of the proposed relationships with the impinging angle, *θ*, i.e., neither the tangent rule, *β*_t_ = arctan (1/2·tan*θ*) nor the cosine rule, *β*_*c*_ = *θ* − arcsin[(1 − cos*θ*)/2], but fell in between *β*_t_ and *β*_c_.

In addition to the relatively large tilting angle of AgNRs, we also find the influence of substrate temperature on the tilting angle. Even though no noticeable change was observed in this work, the observation in a previous report clearly shows that the tilting angle of AgNRs decreases as the substrate temperature is lowered [25]. The tilting angle of the AgNRs, fabricated by an e-beam evaporator with 86° of deposition angle, was observed to decrease by ~2° when the substrate temperature was lowered from room temperature to 140 K [25]. Our observation of no noticeable variation of the tilting angle in the whole temperature range, 10–70 °C can be interpreted as the variation of the tilting angle was too small to be observed clearly.

Directional diffusion effect out of the two diffusion effects is essential for the understanding of the tilting angle. The directional diffusion effect is that adatoms show overall drift parallel to the impinging direction due to the conservation of parallel momentum [11, 26]. Even if the collective drift of the adatoms is small, it can change the tilting angle. It is known that the directional diffusion effect is determined by molecular dynamic properties, such as potential barrier and substrate temperature, but quantitative understanding is not given until now. The other mechanism is the random diffusion or surface diffusion effect. It represents the adatoms’ migration behavior on the metal surface which is conducted via hopping activated by the lattice thermal energy prior to the arrivals of the next atoms. The random diffusion effect is well understood.

The specific tilting angle of AgNRs and its dependence on the substrate temperature mentioned above can be understood by the two diffusion effects. The aforementioned behaviors of the tilting angle follow general observations. That is to say, as random diffusion becomes larger, the tilting angle moves toward the vapor incidence direction. And it is well known that the small energy barrier of Ag crystal and the increase in the substrate temperature elevate the random diffusion effect, which leads to the observations. However, the tilting angle is determined by complicated interplay between the two diffusion effects, and so detailed understanding cannot be obtained yet.

Second, the morphological properties of AgNRs, such as diameter, density, and length, dependent on the substrate temperature as well as the existence of a critical temperature for the AgNR array formation can be explained by the random diffusion effect. Even though the high mobility of Ag adatoms has been attributed to the unique morphological properties of AgNR arrays in previous reports [10–12], more quantitative understanding is still needed. For this purpose, we studied the random surface diffusion effect in AgNR deposition by using the thermodynamic properties of the Ag atom and the theory of Abelmann et al. [11, 27].

The random diffusion distance, *Λ*, can be expressed as

1 $$ \varLambda =\frac{1}{2}{a}_{\mathrm{h}}\sqrt{\frac{\tau_{\mathrm{m}}}{\tau_{\mathrm{h}}}} $$

where *a*_h_ is the atomic radius, *τ*_m_ the time for the growth of one atomic layer, and *τ*_h_ the mean time between hops. And *τ*_h_ is determined by the film temperature, *T*_f_, the energy needed for one hop, *E*_h_, and the lattice vibration frequency, *ω*, as

2 $$ {\tau}_{\mathrm{h}}=\frac{1}{\upomega} \exp \left(\frac{E_{\mathrm{h}}}{k{T}_{\mathrm{f}}}\right) $$

It is known that the least energy needed for surface diffusion or hopping, *E*_h_, is about one fifth of the escape energy which is the energy needed for an atom to escape from the surface or evaporate. The escape energy is equal to the change of enthalpy which is related to the change of the Gibbs free energy by a thermodynamic equation. So, the escape energy dependent on temperature can be obtained by using the related thermodynamic equations and the Gibbs free energy which can be obtained by using the known vapor pressure data dependent on temperature. Following the above method, we calculated the temperature-dependent enthalpies of Ag, Co, Fe, Cr, and Ni atoms in the region from 0 to 2000 °C by using the known vapor pressure data [27] and found that the values of Co, Fe, Cr, and Ni atoms are almost the same as those given in previous reports with differences smaller than 10 % [11]. We note that the escape energy of Ag is much smaller than those of the other atoms by ~30 %. Based on the experimental parameters (*a*_h_ = 0.125 nm, *τ*_m_ = 0.25 s, *ω* = 10^13^ Hz (assumed), etc.), the random diffusion distance, *Λ*, was calculated with respect to temperature.

**Fig. 3:**
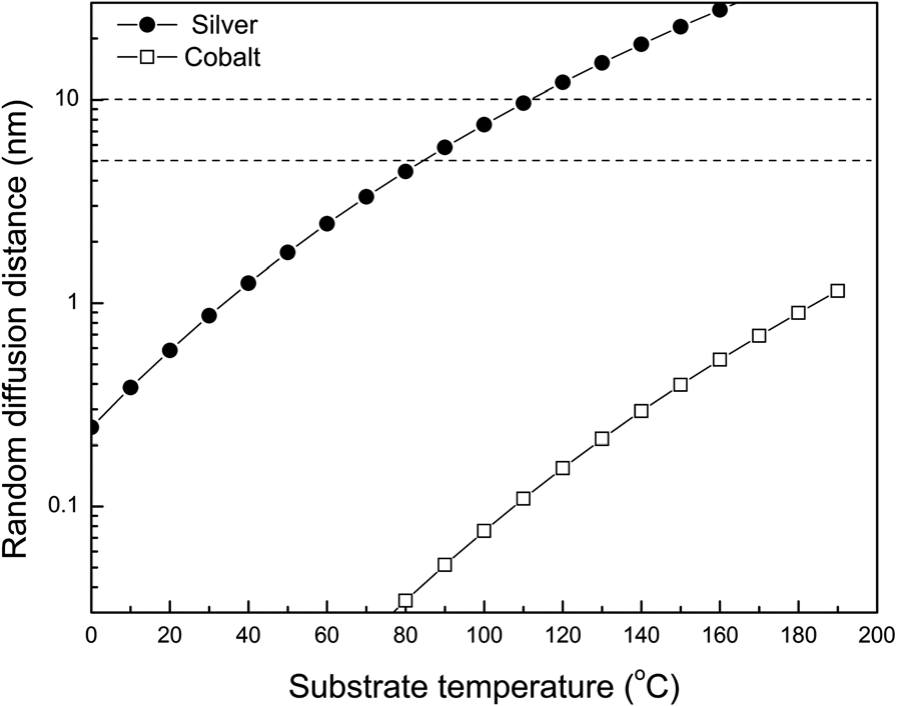
Calculated random diffusion distances of Ag and Co adatoms with respect to substrate temperature using vapor pressure data

Figure 3 shows the random diffusion distances of two representative metals, Co and Ag, with respect to the substrate temperature. From the graph, it is found that the diffusion distance of the Ag adatom is longer than that of the Co adatom by about two orders of magnitude at any temperature from 0 to 200 °C due to the difference of *E*_h_. The diameters of nuclei, formed by islandization mechanism in the early stage of the O.A.D. process, were observed as 10–30 nm and the gaps between the nuclei smaller than 10 nm when the deposition amount of Ag was ~5 nm in the QCM thickness monitor. Reminding these features of the nuclei as an early condition, the random diffusion is thought to influence the columnar growth significantly, especially in the early stage. When the diffusion length is larger than 5 nm, the coalescence between neighboring nuclei may increase so much as to inhibit the formation of Ag nanorods. So, the critical substrate temperature for the nanorod array formation is expected to be ~90 °C for Ag and ~300 °C for Co and many other metal atoms from Fig. 3. Considering the uncertainties of the calculated diffusion lengths and the least diffusion lengths needed for the widespread coalescence of the nuclei, the random diffusion effect seems to explain the observed critical temperatures for the formation of nanorod arrays of Ag, Co, Fe, etc. very well [11, 28].

Furthermore, the influence of the substrate temperature on the other morphological properties, such as diameter, length, and density, can be explained by the random diffusion effect. The nuclei automatically prepared in the early stage of the deposition process will continue expanding owing to the random diffusion effect. Coalescence between small nuclei will follow depending on the scale of the random diffusion effect and the gap size between the particles. The sizes and the density of the nuclei after the expansion and the coalescence will determine the diameter and the density of AgNRs afterwards. The length of nanorods can also be influenced by the random diffusion effect since the expansion and the coalescence processes will reduce the growth rate of AgNRs. The random diffusion distances of Ag with respect to the substrate temperature in Fig. 3 will give us deep insight into the temperature dependence of the morphologies. For instance, the bundling between AgNRs is more apparent at low temperature. Noting that the diameters of identical rods are relatively thin at low temperature, this also can be explained as the small gaps between the thin rods formed in the early stage at low temperature give more chance to bond each other.

**Fig. 4:**
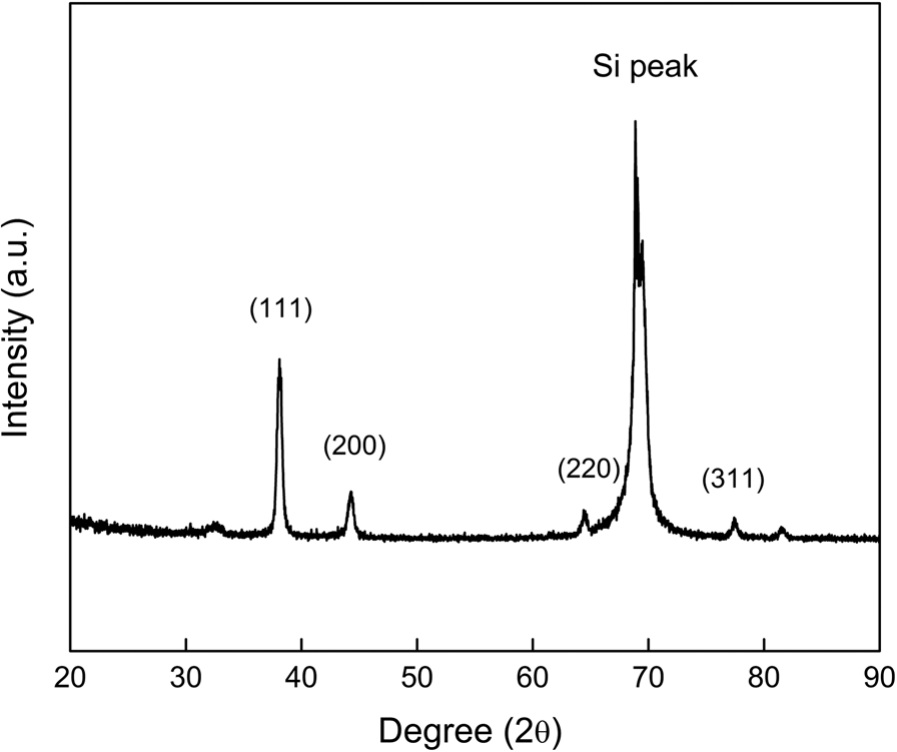
X-ray diffraction pattern of a AgNR array film on silicon wafer

The crystallographic structure of the AgNR array was also characterized by XRD. The XRD pattern in Fig. 4 clearly illustrates that the fabricated AgNR film is crystalline. The diffraction peaks found at 2*θ* = 38.10, 44.22, 64.50, and 77.40 are indexed (*Miller indices*) as (111), (200), (220), and (311) planes, respectively, which correspond to Bragg’s reflection of face-centered cubic (fcc) Ag [29–31]. Among these, dominant out-of-plane growth direction was along the (111) crystal orientation while the others were found to be present with less intensity, which is supported by a similar observation noted by Khare and coworkers [32]. Since no spurious diffractions were found, the fabricated Ag nanorods are proved to be free of crystallographic impurities [33].

### SERS Characterization of AgNR Array Films

**Fig. 5:**
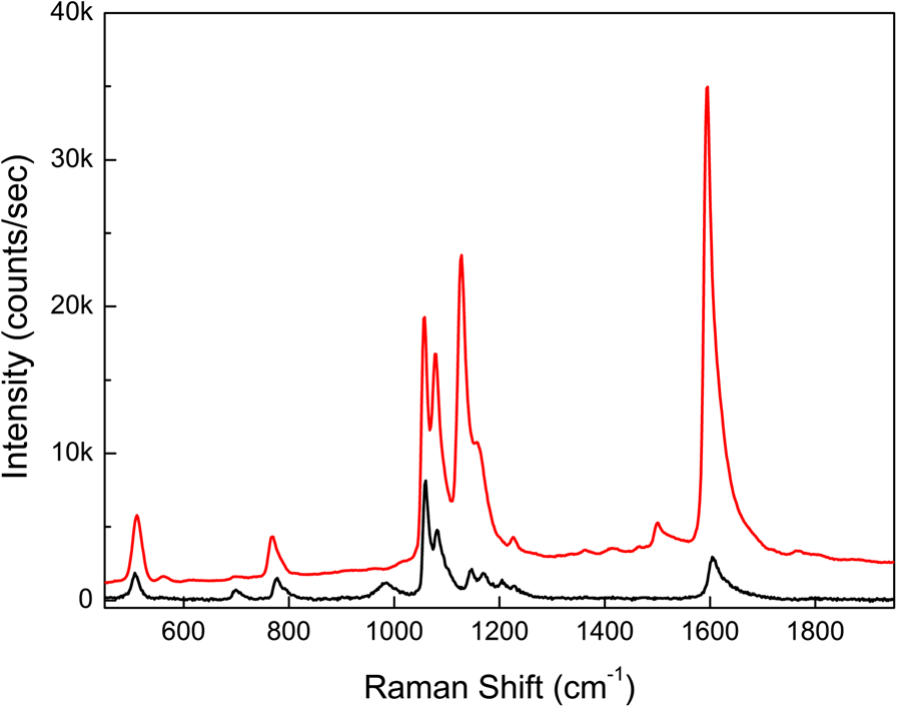
SERS spectrum of benzenethiol SAM on an AgNR array film (*red*) and Raman spectrum of neat benzenethiol (*black*)

Figure 5 shows the SERS spectrum (red line) of the SAM of benzenethiol on the AgNR array film (Fig. 2d) and the conventional Raman spectrum (black line) of neat benzenethiol (95 %) contained in an optical quartz cell of 1-mm path length. For better comparison, the Raman spectrum of neat benzenethiol was magnified ten times. The wavelength of the pump laser was 532 nm and the input power on the samples was 10 mW. The beam diameter of the pump laser at focus was ~30 μm (FWHM) and the acquisition time was 1 s in all the measurements.

When SERS films are functionalized by probe molecules, their E.F.s are calculated as

3 $$ \mathrm{E}.\mathrm{F}.=\frac{I_{\mathrm{SERS}}/{I}_{\mathrm{bulk}}}{N_{\mathrm{SAM}}/{N}_{\mathrm{bulk}}} $$

where *I*_SERS_ and *I*_bulk_ are the intensities of one Raman band measured in a SERS-active medium and bulk state, respectively. *N*_SAM_ and *N*_bulk_ are the numbers of probe molecules in SAM and bulk state contributing to the Raman signal, respectively.

In this work, *I*_SERS_/*I*_bulk_ was ~120 in the Raman band near 1600 cm^−1^ (see Fig. 5). Even though the morphology of our AgNR array film is far from flat, we used the well-known number density of SAM of benzenethiol on a flat Au surface [34], 4.7 molecules/nm^2^, to obtain the value of *N*_SAM_. This was not only for simplicity but also for the E.F. to express the sensitivity or detecting capability of the SERS film explicitly. So, *N*_SAM_ was estimated to be ~4 × 10^9^. *N*_bulk_ is determined by the number density multiplied by the focal volume contributing to the signal intensity. Since the focal volume of our Raman spectrometer is ~3 × 10^5^ μm^3^, *N*_bulk_ was estimated to be ~2 × 10^15^. As a result, the E.F. of the AgNR array film was estimated to be ~6 × 10^7^ using Eq. (3).

**Fig. 6:**
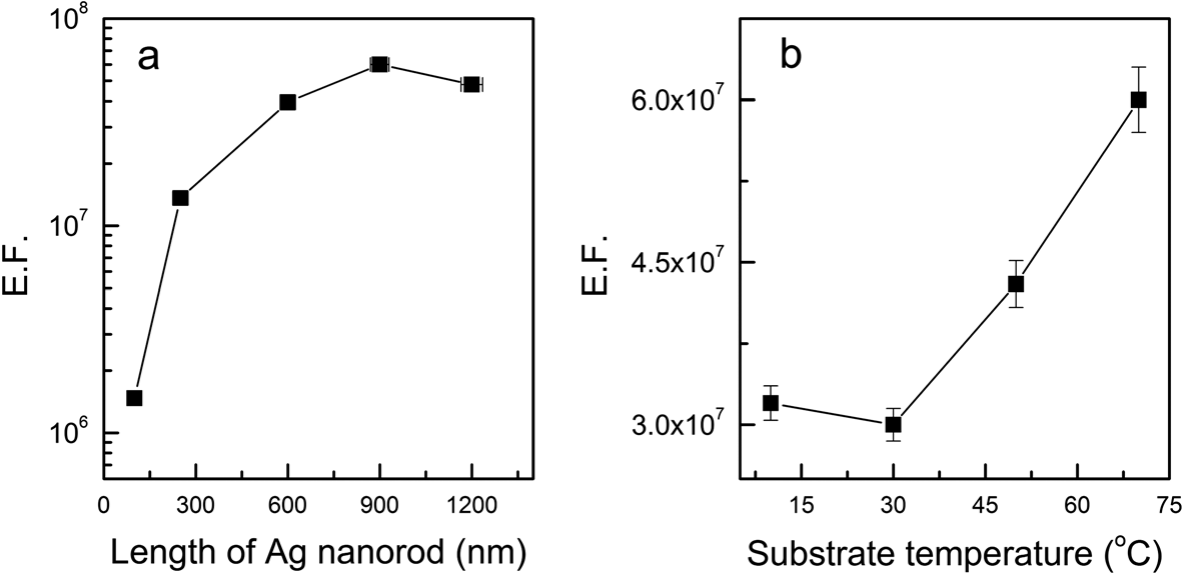
Measured E.F.s dependent on nanorod length (**a**) and substrate temperature (**b**)

The correlation between the E.F. and the morphology of the AgNR array was also investigated. Figure 6 shows the E.F.s of AgNR arrays of different rod lengths (a) and different substrate temperatures during evaporation (b). The substrate temperature during evaporation for the AgNR arrays in Fig. 6a was 70 °C. The wavelength of the pump laser was 532 nm, and the input power of the pump laser at the samples was 10 mW. From Fig. 6a, it is found that the optimum nanorod length is ~820 nm. And the variation of the E.F. can be characterized by three sections. In the first zone, where the nanorod length is shorter than 250 nm as well as the aspect ratio is smaller than 4.5, the growth rate of the E.F. is much higher than that of the nanorod length. In the second zone, located in the range of 250–820 nm, the E.F. linearly increases with respect to the nanorod length. In the third zone, where the nanorod length is longer than 820 nm, the E.F. decreases as the length of nanorods increases.

In the first zone, the Ag thin film shows a kind of morphological change from AgNP to AgNR during the deposition process, to which the dramatic increment of the E.F., larger than two orders of magnitude, is attributed. This observation cannot be explained by general understanding of AgNP or AgNR SERS substrates, which implies that some hidden mechanism should have played a crucial role for the appearance of the huge E.F. in the AgNR arrays. The implication is twofold. First, hot spots or narrow gaps between nanorods showed up more and more as the nanorods grew up. Second, another unknown mechanism played the main role.

Our observation in the second zone, the linear increment of the E.F. with respect to the nanorod length, supports the first scenario, i.e. the hot spot effect since it can be explained by the increase of the number of hot spots in longer nanorods. However, it is not easy to find appropriate sites for such kind of hot spots in the relevant SEM images (Fig. 2). In addition, our observation in Fig. 6b, the AgNR arrays with higher nanorod density (fabricated at low substrate temperature) showed lower E.F. than the films with lower density (fabricated at high temperature), also contradicts the first scenario based on the hot spot mechanism. That is to say, more efforts are needed to reveal the mechanism for the observed huge E.F. of the AgNR arrays, which called for the theoretical study of the next section.

The decrease of the E.F. in the third zone seems to have resulted from the degradation of the AgNR array with extended deposition process and the limited thickness of AgNR array contributing to SERS signal due to the attenuation of the incident light propagating in the AgNR arrays.

When the pump laser wavelength was changed to 633 and 785 nm, the E.F. and its dependence on morphology were proved to be almost the same as those described above. Reminding the differences in the scheme of analysis, such as kind of probe molecules, SAM or physical adsorption for probe molecules on the surface, and incidence angle of the pump laser, the AgNR array films seem to have similar SERS properties to those of the films fabricated by e-beam evaporation [10].

### Theoretical Calculation

**Fig. 7:**
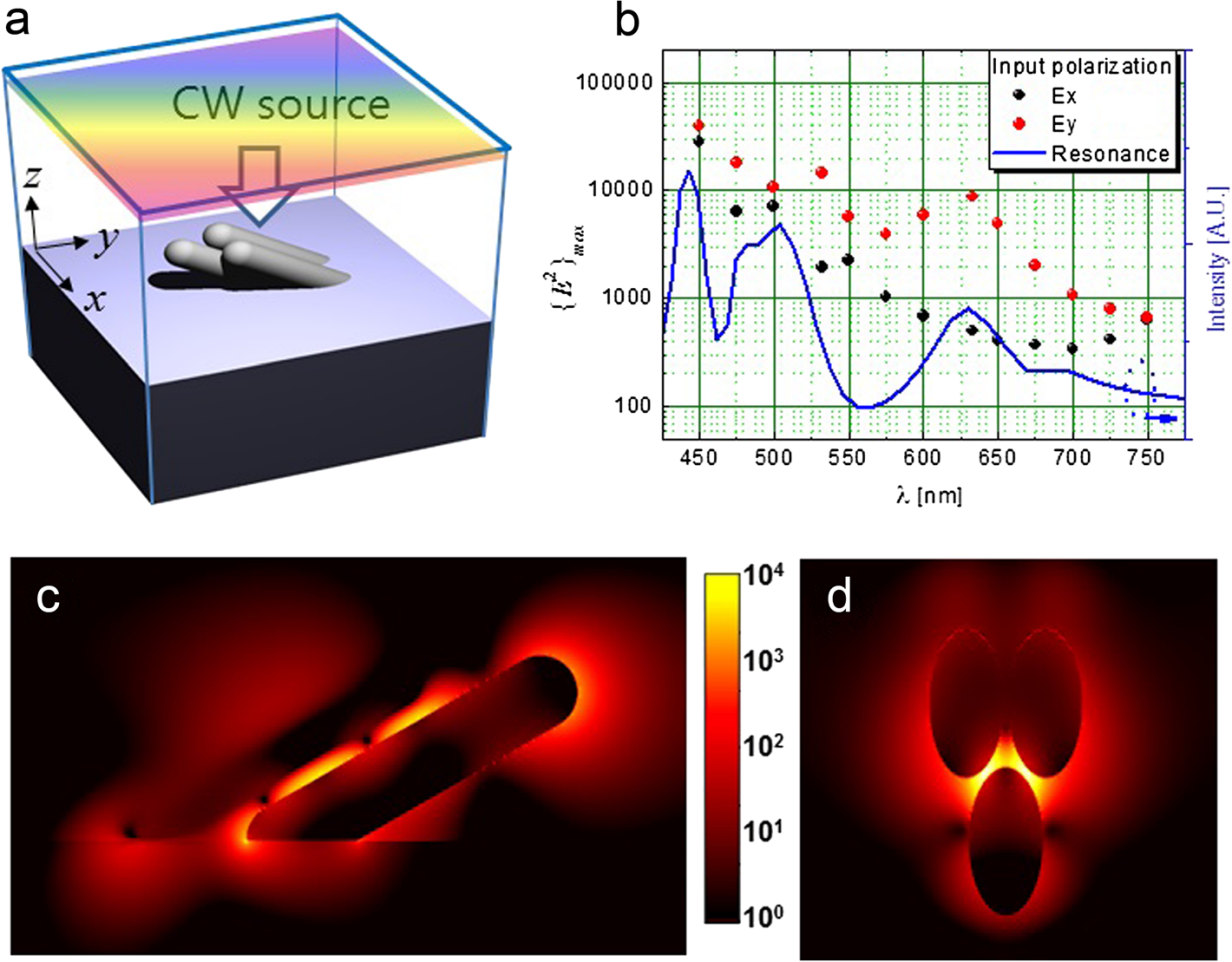
FDTD simulation results. **a** Simulation structure. **b** Local maximum field intensity, {*E* ^2^}_max_, as a function of wavelength and resonance spectrum. **c** *yz* cut-view and (**d**) *xy* cut-view of the *E* ^2^ field at 633 nm in the normal incidence of *E*_*y*_-polarized plane wave

In order to understand the physical origin of the high E.F. of the AgNR array, a theoretical study was performed based on numerical calculation with three-dimensional FDTD method [35]. Figure 7a shows the schematic view of the calculation structures. The 60°-tilted three Ag nanorods are closely positioned on top of the SiO_2_ substrate with a very small gap, 3 nm. The length, *l*, and the radius, *r*, of the nanorod are 200 and 25 nm, respectively. In determining the calculation structure, the authors considered many kinds of geometry to reflect the real morphology as much as possible. For example, different gap sizes between rods, rods with bumps, and branch structure were modeled and tried for calculations. As a result, such huge E.F. up to 10^8^ was found only when narrow gaps smaller than 3 nm exist irrespective of the geometrical scheme. Therefore, the tilted three nanorods with 3-nm gap was selected as a representing structure to reproduce the experimental observation theoretically.

In the simulation, Ag was modeled using the Drude equation in all visible range [36]. We note that the minimum spatial size (grid size) should be small enough to resolve the field distribution in spatially discrete numerical simulation. But a 1-nm resolution in space was maintained in this study considering the limited computational power. We expect that if the resolution is finer than 1 nm, the E. F. will be increased further.

First, we checked the optical resonance in our model system by one-shot calculation for the broadband investigation. The blue line in Fig. 7b is the resonance spectrum which was obtained from the Fourier transformation of the recorded field at the center of the gap among the nanorods after the *E*_*y*_-polarized pulse excitation. The local intensity maximum shows a broad surface plasmon (SP) resonance spectrum with three noticeable resonant peaks around 440, 500, and 630 nm. In order to understand the origin of these resonances, we excited the SP modes by using the continuous-wave (CW) incident field of 633 nm as shown in Fig. 7c, d. The field distribution shows a distinct mode structure along the nanorod direction as well as strong concentration in the gap region. From the results, the resonance modes are found to be related with the coupled longitudinal SP modes of the Ag nanorods originating from the small gap.

The E.F. of the Raman signal was also numerically estimated by obtaining the local maximum of the electric field intensity as a function of wavelength. In this calculation, CW sources were used and the local intensity maximum was obtained after stabilizing the field intensity. Here, the input intensity of the electric field was fixed as 1. Black and red circles in Fig. 7b represent the local intensity maxima at each wavelength in respective input polarizations (*E*_*x*_, *E*_*y*_). The overall shapes resemble the aforementioned resonance spectrum (blue line) especially in the *E*_*y*_ polarization. The intensity maximum in the *E*_*y*_ input polarization is larger than that in the *E*_*x*_ input polarization in the whole spectral range because the *E*_*y*_-polarized light is more appropriate for the coupling with the longitudinal SP mode in the Ag nanorods. The intensity maximum at 532 nm is ~10^4^ with the corresponding E.F. of the Raman signal ~10^8^. Even though the maximum E.F. should be larger than the average E.F. in the whole surface area, this calculation result seems to reproduce the experimental value in one order of magnitude.

## Conclusions

In this work, AgNR arrays of various morphologies were fabricated by thermal evaporation at oblique angle. The morphology of the AgNR array, delicate to the substrate temperature even in the low temperature region, and the critical temperature ~90 °C for the formation of the AgNR array seem to originate from the large diffusion effect of the Ag adatom. From SERS characterization, the highest E.F. of the AgNR array films was found to be as large as 6 × 10^7^. With the help of the numerical calculation performed, hot spots or coupled longitudinal SP modes excited in the narrow gaps are attributed to the huge E.F. of the AgNR array. But there are experimental observations in contradiction to the hot spot effect. Sufficient understanding on the physical origin of the huge E.F. will help promote the E.F. of the AgNR array further. Except for the morphological variations dependent on the substrate temperature, the overall characteristics of our AgNR array films are very similar to those of the Ag films fabricated by e-beam evaporation [11].

From this work, it was proven that thermal evaporation can be a feasible alternative to e-beam evaporation for the fabrication of the AgNR array by O.A.D. And the important role of the diffusion effect in the AgNR array formation, revealed by this study, proposes the substrate temperature control for AgNR films of new morphology. It is expected that this work will contribute to the development of highly sensitive SERS films and provide more opportunities to researchers who urgently need novel SERS films. We are currently planning to apply the AgNR array films to highly sensitive multiple-gas sensing through surface functionalization by various thiol molecules.

## Abbreviations

AAO: anodic aluminum oxide
AFM: atomic force microscopy
AgNR: silver nanorod
*a*_h_: atomic radius
CCD: charge-coupled device
CW: continuous wave
E.F.: enhancement factor
*E*_h_: energy needed for one hop
FDTD: finite-difference time-domain
FIB: focused ion beam
FWHM: full width at half maximum
O.A.D.: oblique angle deposition
QCM: quartz crystal microbalance
SAM: self-assembled monolayer
SEM: scanning electron microscopy
SERS: surface-enhanced Raman scattering
SP: surface plasmon
*T*_f_: film temperature
XRD: X-ray diffraction
*Λ*: random diffusion distance
*τ*_h_: mean time between hops
*τ*_m_: time for the growth of one atomic layer
*ω*: lattice vibration frequency
